# Free-Space Nanometer Wiring via Nanotip Manipulation

**DOI:** 10.1038/srep13529

**Published:** 2015-08-26

**Authors:** Tokushi Kizuka, Shin Ashida

**Affiliations:** 1 Division of Materials Science, Faculty of Pure and Applied Sciences, University of Tsukuba, Tsukuba, Ibaraki 305-8573, Japan

## Abstract

Relentless efforts in semiconductor technology have driven nanometer-scale miniaturization of transistors, diodes, and interconnections in electronic chips. Free-space writing enables interconnections of stacked modules separated by an arbitrary distance, leading to ultimate integration of electronics. We have developed a free-space method for nanometer-scale wiring on the basis of manipulating a metallic nanotip while applying a bias voltage without radiative heating, lithography, etching, or electrodeposition. The method is capable of fabricating wires with widths as low as 1–6 nm and lengths exceeding 200 nm with a breakdown current density of 8 TA/m^2^. Structural evolution and conduction during wire formation were analyzed by direct atomistic visualization using *in situ* high-resolution transmission electron microscopy.

For stripes, i.e., wire formations attached to substrates, the width has been reduced to 60 nm using chemical etching of ion tracks followed by successive deposition of copper^1^,^2^,^3^,^4^ and to 8 nm using translating nanowire (NW) patterns to substrates without etching and lift-off^5^. Thus, the scales of transistors, diodes, and interconnections have reached comparable levels on a substrate. For further integration of electronic chips, three-dimensional laminated implementation has been anticipated; free-space wiring enables interconnections of nanometer-thick stacked modules^1^,^2^,^3^. Free-standing copper and platinum wires with widths of 150–200 nm have been fabricated by electrochemical deposition using pipette probes^6^,^7^. Omnidirectional printing using silver nanoparticle inks has spanned microelectrodes using flexible silver wires having widths of ~2 μm^8^. Meniscus-confined three-dimensional electrodeposition enables direct writing of copper wires with widths of 740 nm^9^. By applying the same method to polymers, the wire width can be decreased to ~50 nm^10^. Various shaped gallium wires of similar widths, i.e., 80 nm, can be fabricated by focused-ion-beam chemical deposition^11^. Thus, downsizing free-space wiring to several nanometers, which is comparable to the sizes of transistors, diodes, and stripes on substrates, realizes the ultimate integrated electronics on the basis of laminated implementation.

Thin free-space metallic wires with typical widths of several nanometers have been studied in relation to ballistic conduction, and the resulting quantization of conductance is defined by a quantum unit (G_0_ = 2*e*^2^/*h*, where *e* is the charge of an electron and *h* is Planck’s constant)^12^,^13^,^14^. In this field, such NWs have been fabricated by elongation of nanometer-sized contacts, i.e., the contact of two nanotips and subsequent tensile deformation^15^,^16^. Using the mechanical breakdown method, wires of noble metals have reached lengths of several nanometers. Thus, by selecting conditions such as metallic species, applied voltage, and writing speed, this method is expected to fabricate longer metallic NWs. In this report, we demonstrate the fabrication and wire bonding of zinc (Zn) NWs via nanotip manipulation while applying bias voltages.

## Results

### The formation process of Zn NWs

**Figure 1:**
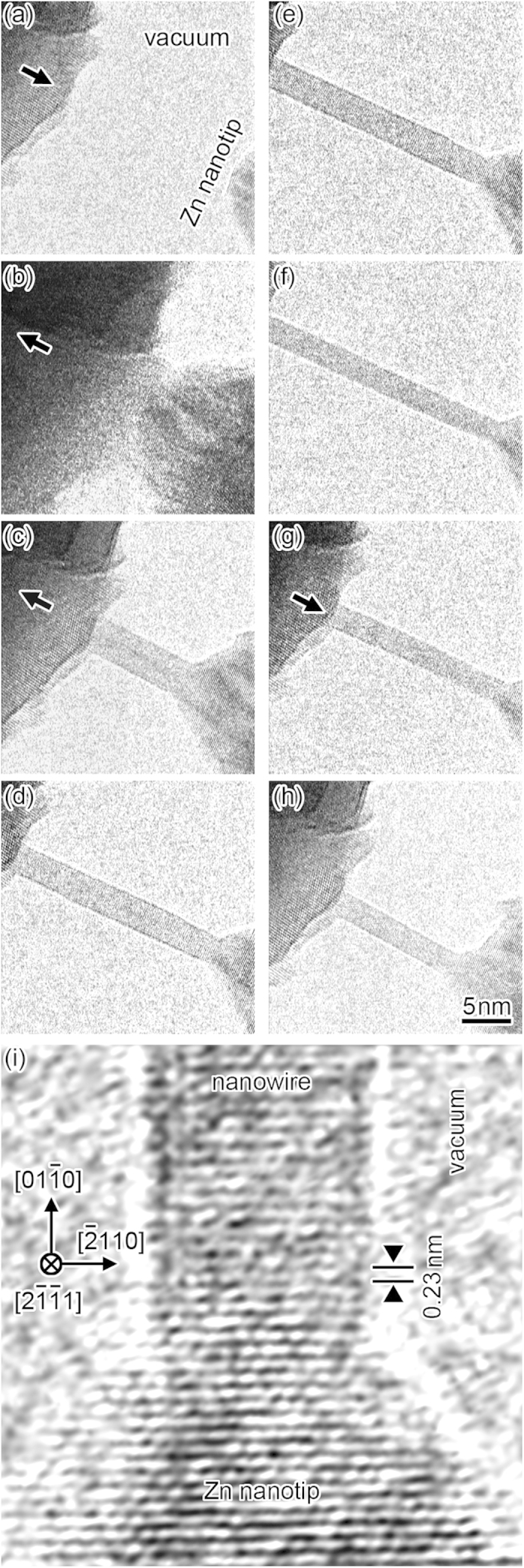
(a–h) Time sequence high-resolution TEM images during the formation of a Zn NW while applying a bias voltage of 15 mV. The nanotip at upper left was moved in the direction indicated by the bold arrow. The sequence is grouped as (**a**,**b**) the process for contacting the two nanotips, (**c**–**f**) the process of forming the NW by elongation of the nanocontact, and (**g**,**h**) the process of compressing the NW. (**i**) High-resolution image of the growth site of the NW is shown in (**e**).

Figure 1 shows a time sequence of high-resolution transmission electron microscopy (TEM) images during the formation process of a Zn NW while applying a bias voltage of 15 mV (Supplementary Movie 1). The upper left and lower right dark regions of each frame in Fig. 1 are the positively and negatively biased Zn nanotips, respectively. First, the two nanotips were separated in the vacuum (Fig. 1a). Next, the nanotip at the upper left was maneuvered toward the other nanotip, resulting in contact of both nanotips (Fig. 1b). Then, the nanotip at the upper left was pulled back in the opposite direction. Using this separation, a NW with a width of 4 nm was formed between the nanotips (Fig. 1c). The NW grew by subsequent retraction of the nanotip at the upper left (Fig. 1d–f). Figure 1(i) shows a high-resolution TEM image of the growth site of the NW, which enlarges the connecting region of the NW and the nanotip at the lower right in Fig. 1e. A 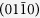 lattice of Zn with a hexagonal-close-packed structure appears on both the NW and the nanotip, implying that a single-crystal Zn NW grew along the same direction of the nanotip, i.e., the direction parallel to the long axis of the NW (the 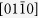 direction). The length of the NW reached 25 nm (Fig. 1f).

**Figure 2:**
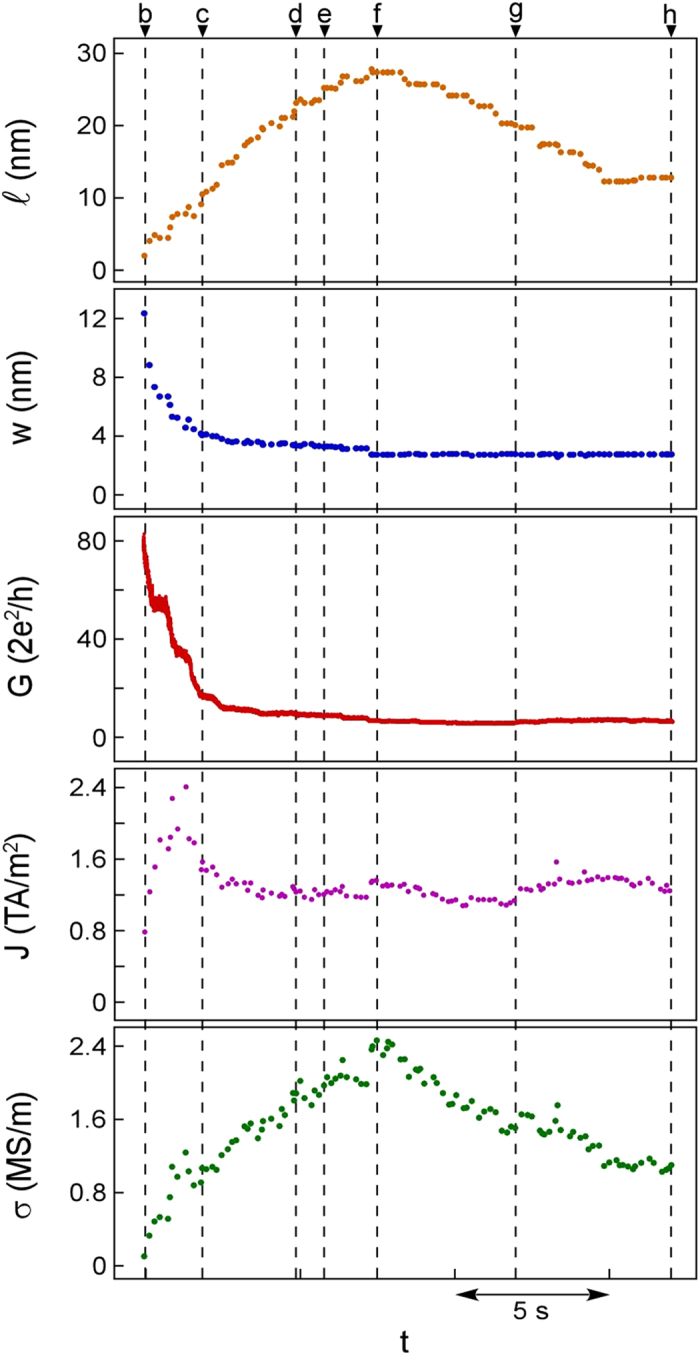
Changes in length (*ℓ*), minimum cross-sectional width (*w*), conductance (*G*), current density (*J*), and electrical conductivity of the Zn NW in Fig. 1 as functions of time (*t*). Times indicated by b–h correspond to recording times of the images in Fig. 1b–h, respectively. Conductance is expressed in units of G_0_ = 2*e*^2^/*h*, where *e* is the charge of an electron and *h* is Planck’s constant.

Subsequently, to observe compression of the NW, the nanotip at the upper left was moved towards the other nanotip (Fig. 1g,h). The NW was shortened by this compression while showing neither bending nor fracture; the portion of the NW around the connecting region was absorbed into the nanotip. Thus, the length of a NW can be controlled by mechanical operations. The length, minimum cross-sectional width, conductance, current density, and electrical conductivity of the NW during the formation process in Fig. 1 are shown in Fig. 2. The electrical conductivity was estimated after the straight NW with a homogeneous width emerged (time b in Fig. 2). As the separation distance increased from time b to time f, the length of the NW increased and the width decreased. During this period, the electrical conductivity increased to 2.4 MS/m, which is approximately 14% of that of bulk Zn crystals, although the current decreased. After time f, the NW was shortened from 27 nm to 12 nm by compression (Fig. 1f–h). During this period, the width remained at ~3 nm and the conductance was almost unchanged. Thus, conductance was determined by width and not by length. During the shortening of the NW, the electrical conductivity decreased to 1.1 MS/m. Assuming that the cross section of the NW was circular, the current density was estimated to be 1.2 TA/m^2^, which is approximately a half of that of Zn nanocontacts estimated in an ultrahigh vacuum using the Sharvin formula^17^ and comparable to the failure current density of copper and gold wires having widths of 100 nm (1.5 TA/m^2^ and 1.2 TA/m^2^, respectively)^18^,^19^. The variation in conductance resembles that at the minimum cross-sectional width. Thus, this also shows that the conductance is governed by minimum cross-sectional width and not by length.

**Figure 3:**
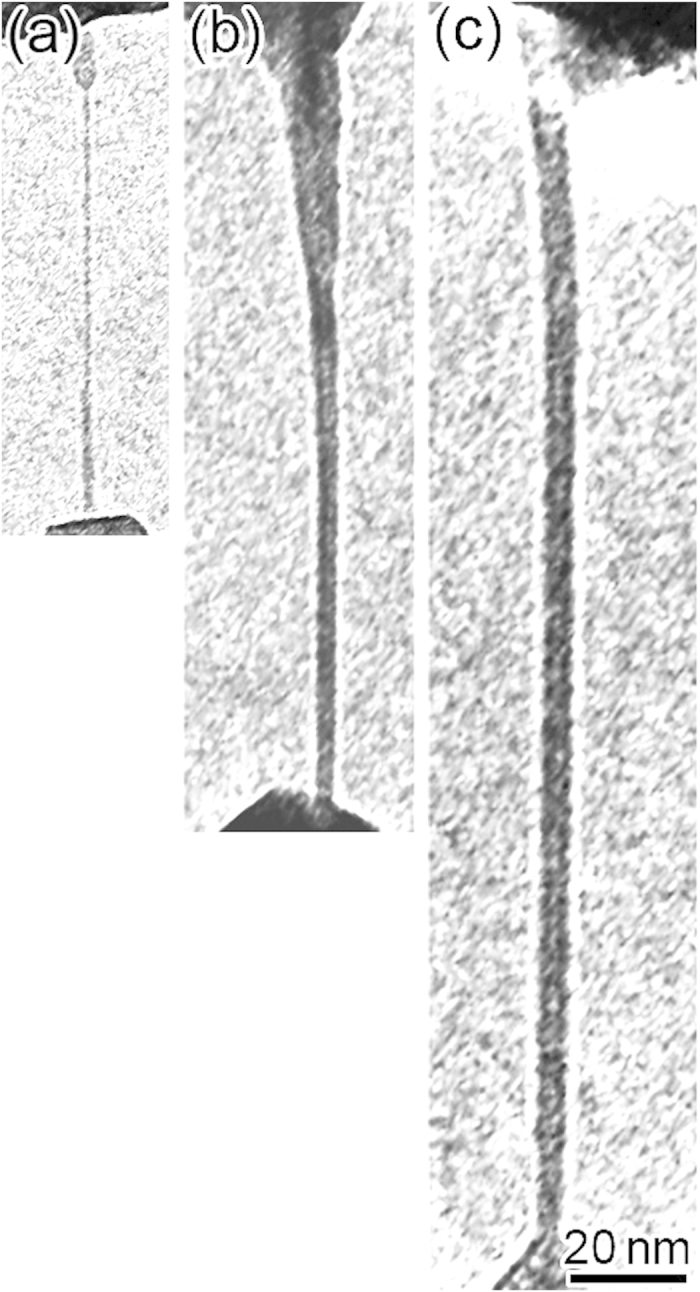
TEM images of Zn NWs produced at applied voltages of (a) 0 mV, (b) 26 mV, and (c) 45 mV. Wire lengths and widths were (**a**) 84 nm and 1 nm, (**b**) 133 nm and 4 nm, and (**c**) 225 nm and 6 nm, respectively.

Figure 3 shows TEM images of Zn NWs with maximum wire lengths at several applied voltages. The maximum length increased with applied voltage: 84 nm at 0 mV, 133 nm at 26 mV, and 225 nm at 45 mV. The wire width also increased: 1 nm at 0 mV, 4 nm at 26 mV, and 6 nm at 45 mV. Note that NWs were produced even at zero voltage; the length, 84 nm, was the limiting one when a bias voltage was not applied. The maximum formation speed of NWs at 45 mV was 114 nm/s.

### Electromigration due to bias application

To investigate the effects of bias voltage on structural dynamics of stress-free states, we fixed the two nanotips connecting a NW and observed structural dynamics while applying bias voltages (Supplementary Fig. S1). In particular, we investigated variations in current and current density of the Zn NW as the bias voltage was changed (Supplementary Fig. S2). When the applied voltage was less than 100 mV, no structural changes were observed in the NW (Supplementary Fig. S1(a)). In this voltage region, the current increased linearly to 1000 μA against the voltage (Supplementary Fig. S2). The current density also increased linearly against voltage. When the bias voltage reached 100 mV, a surface step emerged (Supplementary Fig. S1(b)) and the current density increased to 11 TA/m^2^ (point b in Supplementary Fig. S2). At higher voltages, the width of the NW near the negatively biased electrode decreased owing to electromigration, and a clear crystal habit appeared (Supplementary Fig. S1(c))^20^; atoms in the NW migrated along the direction from the negatively biased to the positively biased side. During this thinning, the current density at the minimum cross section increased, although the current rapidly decreased to 400 μA (point c in Supplementary Fig. S2). For other NWs of various widths, we investigated the critical current density for electromigration (Supplementary Fig. S3). The critical current density was 8.1 ± 4 TA/m^2^ when the widths were in the range of 3–11 nm.

### Free-space nanometer-scale wire bonding

**Figure 4:**
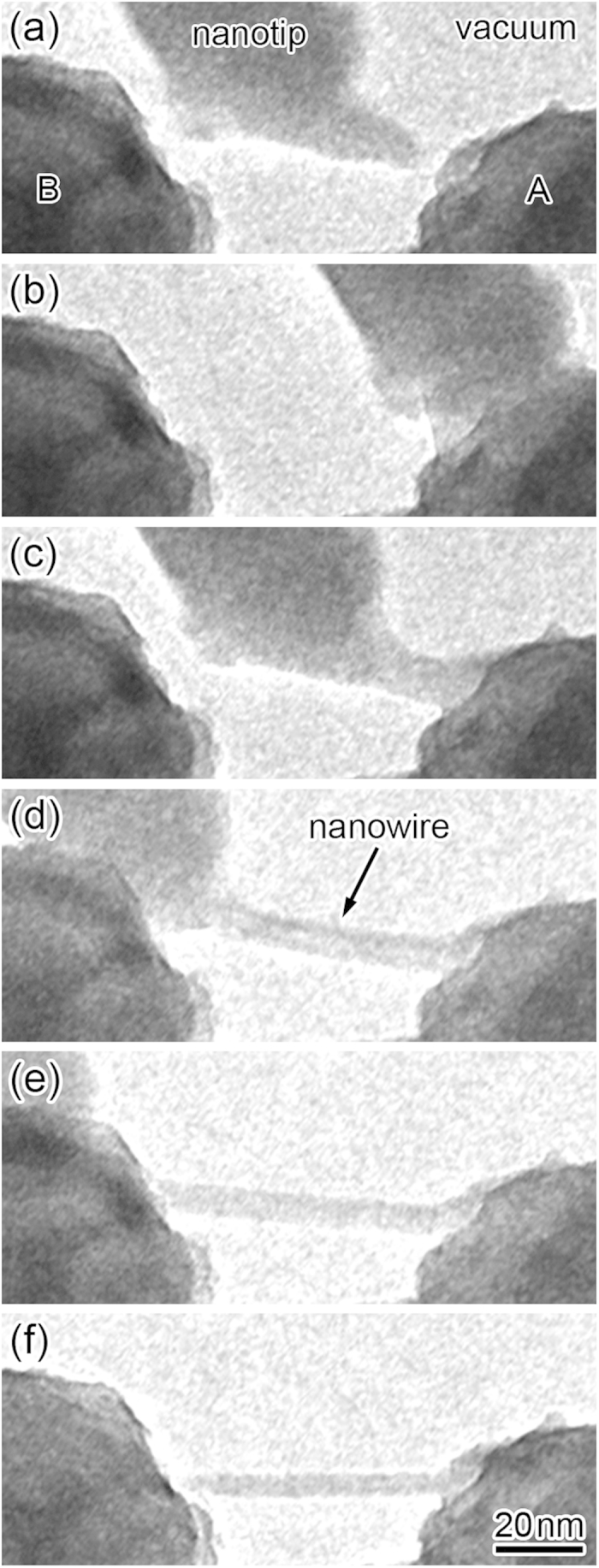
Time sequence TEM images of the NW bonding process using a Zn nanotip between electrodes A (right) and B (left).

Using the wire formation process presented here, we performed free-space nanometer-scale wire bonding, as shown in Fig. 4 (Supplementary Movie 2). As already shown in Fig. 1, a Zn NW was produced by contact between a Zn nanotip and a Zn electrode (electrode A) (right side of Fig. 4a) and subsequent pulling of one nanotip (Fig. 4a–d). Then, the nanotip was contacted with the other Zn electrode (electrode B) (Fig. 4e). Finally, the nanotip was separated from electrode B, resulting in bridging by the NW between the two electrodes (Fig. 4f). The nanotip thinned again by elongation at separation. Thus, wire bonding is repeatable without reformation of the nanotip.

In repeating the wiring process after the formation of NW bridges between electrodes, we moved the nanotip in the direction perpendicular to the wire (Supplementary Fig. S4). When the NW was robustly joined to the electrode, the nanotip separated from the fixed NW by a shift perpendicular to the wire. If the NW was not joined or was only weakly joined to the electrode, the NW separated from the electrode with the nanotip movement. Thus, bridge formation could be confirmed by such observations.

## Discussion

Even at zero voltage, the lengths of growing Zn NWs reached 84 nm and their widths decreased to 1 nm. This maximum length is much higher than that of NWs of other metallic species, which are typically only several nanometers^12^,^13^,^14^,^16^. The deformation behavior of metallic nanocontacts has been investigated on the basis of the slip mechanism, i.e., plastic deformation in both theoretical and experimental work when the applied voltage is zero or is much lower than the critical voltage for electromigration^21^,^22^,^23^,^24^. Elongation of nanocontacts of several nanometers in length can be explained by the slip mechanism. However, in this study, significant growth of NWs 84 nm long was observed at the zero-bias condition (Fig. 3a). Therefore, it is proposed that, in addition to slip events, atomic diffusion accelerated by tensile forces have contributed to the NW growth at the zero-bias condition.

Although Zn showed the maximum wire length in metallic species at zero voltage, the length is smaller than that at biased conditions; the wire length of NWs increased with applied bias voltage. Thus, NW formation was accelerated by electromigration. The growing region of a NW is the region connected between the NW and the negatively biased electrode. Atoms in the growing region are extracted by tensile forces, and atoms in the negatively biased electrode are supplied by electromigration. Because the separation speed of the positively biased electrode increases, the deficit of atoms is not recovered by electromigration, resulting in thinning of the NW and fracture in the growing region. Thus, the maximum speed of NW formation is limited by the applied bias voltage (114 nm/s at 45 mV). Although the present NW formation process resulted from electromigration, the growth mechanism resembles that of the Czochralski method and NW expansion in microcrucible systems, which is based on thermal diffusion at high temperatures^25^.

Since the NW formation observed in this study is attributed to electromigration accelerated by mechanical stress, it is important to consider the theoretical background of both the mechanical and electromigration effects. During electromigration, in which atoms (ions) migrate owing to electron wind forces and electric fields, the migration rate of atoms depends on the bonding nature of each atom positioned at various states. Hence, atoms at surfaces, defects, and deformation regions migrate easily compared with atoms in the defect-free bulk. In this study, stress concentration occurred at the contact regions of nanotips, leading to elastic and plastic deformation. In elastic deformation regions, crystal lattices were distorted and atomic bonding was weakened. In plastic deformation regions, in addition to elastic effects, atomic bonding at slip planes was once separated during slip events. Furthermore, lattice defects such as vacancies and dislocations, and grain boundaries were introduced by slips and atomic bonding at such defects also weakened. Consequently, we inferred that electromigration in the contact regions of nanotips, i.e., the growing regions of NWs, was accelerated by mechanical stress. In particular, when tensile forces acting on the contact region (Fig. 1b–f), the tensile direction was the same as the electromigration direction, resulting in significant acceleration of electromigration. On the other hand, when the NW was shortened by compression (Fig. 1g,h), the direction of atomic movement in the intruded region was opposite of electromigration. Consequently, the acceleration of electromigration at the compression was smaller than that of the tensile condition. Therefore, electromigration feature became different according to the force conditions.

As described above, the acceleration of electromigration by mechanical stress can be qualitatively explained. However, a theoretical quantitative description of atomic motion seems to be difficult. This is because it is difficult to calculate the motions of over 10^4^ atoms that are caused by electron wind forces in fluctuating potentials under mechanical deformation. Therefore, such a theoretical evaluation would extend the current work and will be pursued in the next step of the present experiment. In fact, theoretical work for electromigration was performed in systems smaller than metallic nanocontacts, i.e., in gold single-atom-width wires^26^. The work reports a relationship between acting force and bias voltage; current induces embrittlement of the atomic wires and the tension at fracture decreases with bias voltage. Studies on mechanical behavior of metallic nanocontacts have focused only on elastic and plastic deformations such as slip^21^,^22^,^23^,^24^.

In stress-free or stressless nanocontacts, as shown in Fig. S1, electromigration occurred preferentially at surfaces because the critical voltages for surfaces were lower than those for the bulk^27^,^28^. However, as shown in Fig. 1, high-diffusivity buffer regions formed in stress-concentrated contact regions, and atomic motion was activated over the entire buffer regions. Thus, lattice diffusion, in addition to surface diffusion, was enhanced.

The present method can be applied to conductive species, i.e., metals and semiconductors. The key in this method is control of the activation of atomic diffusion. Control becomes difficult for high-melting-point or tough materials, although atomic diffusion of nanocontacts of such materials is activated by pulsed currents^29^. It is plausible that the formation and bonding of longer NWs is easier for metals that have relatively lower melting temperatures.

In summary, the present method can be used for bonding of the finest wires with widths of less than 6 nm, which is much smaller than wire widths prepared by previous methods. In addition, the present method is based on mechanical operation of a nanotip without radiation heating, and wire width can be controlled by applying a voltage and controlling the manipulation speed of the nanotip. In addition, this process for wire bonding is repeatable without reformation of the nanotip. Thus, controlling the wire bonding is easier than that in previous methods. The NWs fabricated in this study showed single-crystal structures, several nanometer widths, lengths exceeding 200 nm, and a breakdown current density at 8 TA/m^2^. These features are suitable for wire bonding in nanoelectronic applications.

## Methods

To study fabrication and writing of NWs, we used *in situ* high-resolution TEM combined with piezomanipulation of specimens and conductance measurements^30^,^31^. To prepare the nanocontacts, we used piezomanipulation while applying voltages of up to 200 mV to contact a Zn nanotip with an opposing edge surface of a Zn plate having a thickness of 10–20 nm. After realizing the nanocontact, we manipulated the nanotip to elongate the nanocontact to form the NW. We performed a series of these manipulations at ambient temperature in a vacuum of 10^−5^ Pa inside the microscope at the University of Tsukuba. We observed structural dynamics of the procedure *in situ* by lattice imaging using a video capture system with a time resolution of 67 ms. Observations were performed using an acceleration voltage of 200 kV. We measured the current through the NWs by a two-terminal method. We analyzed TEM images and current for every image frame.

## Additional Information

**How to cite this article**: Kizuka, T. and Ashida, S. Free-Space Nanometer Wiring via Nanotip Manipulation. *Sci. Rep.* **5**, 13529; doi: 10.1038/srep13529 (2015).

## Supplementary Material

Supplementary Figures

Supplementary Movie 1

Supplementary Movie 2
