# Supplementary material for: Free-Space Nanometer Wiring via Nanotip Manipulation
Source: Sci Rep. 2015 Aug 26;5:13529. doi: 10.1038/srep13529 (PMC4549688; doi:10.1038/srep13529)
Supplement: Supplementary Figures [file srep13529-s1.docx]

**Supplementary Figures and Movies**

Title: Free-Space Nanometer Wiring via Nanotip Manipulation

Authors: Tokushi Kizuka^*^ and Shin Ashida

Author affiliation: Division of Materials Science, Faculty of Pure and Applied Sciences, University of Tsukuba, Tsukuba, Ibaraki 305-8573, Japan

**Figures**

Figure S1 | Time sequence high-resolution images of the electromigration process in a Zn NW due to application of bias voltages of up to 130 mV. Nanotips at the left and right were biased positively and negatively, respectively. Electromigration starts on the side surface, and a step forms due to the applied bias voltage, as indicated by the arrow in (b).

Figure S2 | Changes in the current (*I*) and current density of the Zn NW as functions of the bias voltage (*V*) during the electromigration process presented in Fig. S1. Times indicated by a–c correspond to the recording times for the images in Figs. S1a–c, respectively. Electromigration started at time b. Crosses indicate fracture.

Figure S3 | Critical current density (*J*) for electromigration plotted as a function of wire width (*w*) for 14 Zn NWs. Current density ranges from 4.1–11.7 TA/m^2^ with an average of 8.1 TA/m^2^.

Figure S4 | TEM images of the nanotip positions manipulated before contact of electrode B (a) and after formation of nanowire and separation from electrode B (b). Images (a) and (b) were observed before Fig. 1(a) and after Fig. 1(f), respectively. Schematic of the nanotip movement (c): contact with electrode B (1), nanowire formation (2), and separation from electrode A (3).

**Movies**

Movie 1 | Movie of the formation of a Zn NW while applying a bias voltage of 15 mV, corresponding to Fig. 1.

Movie 2 | Movie of NW bonding using a Zn nanotip between two electrodes, corresponding to Fig. 4.

Figures


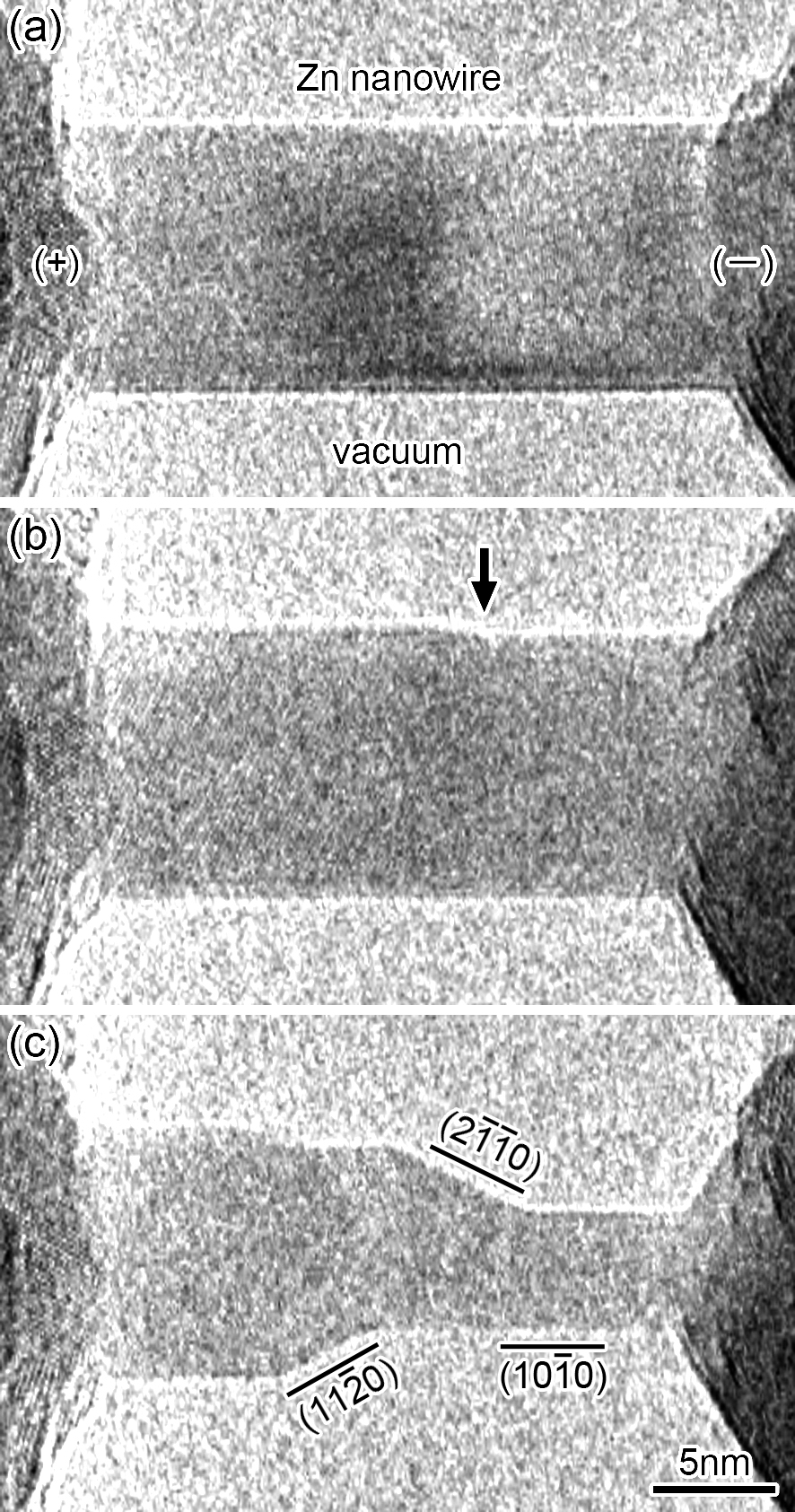


Figure S1


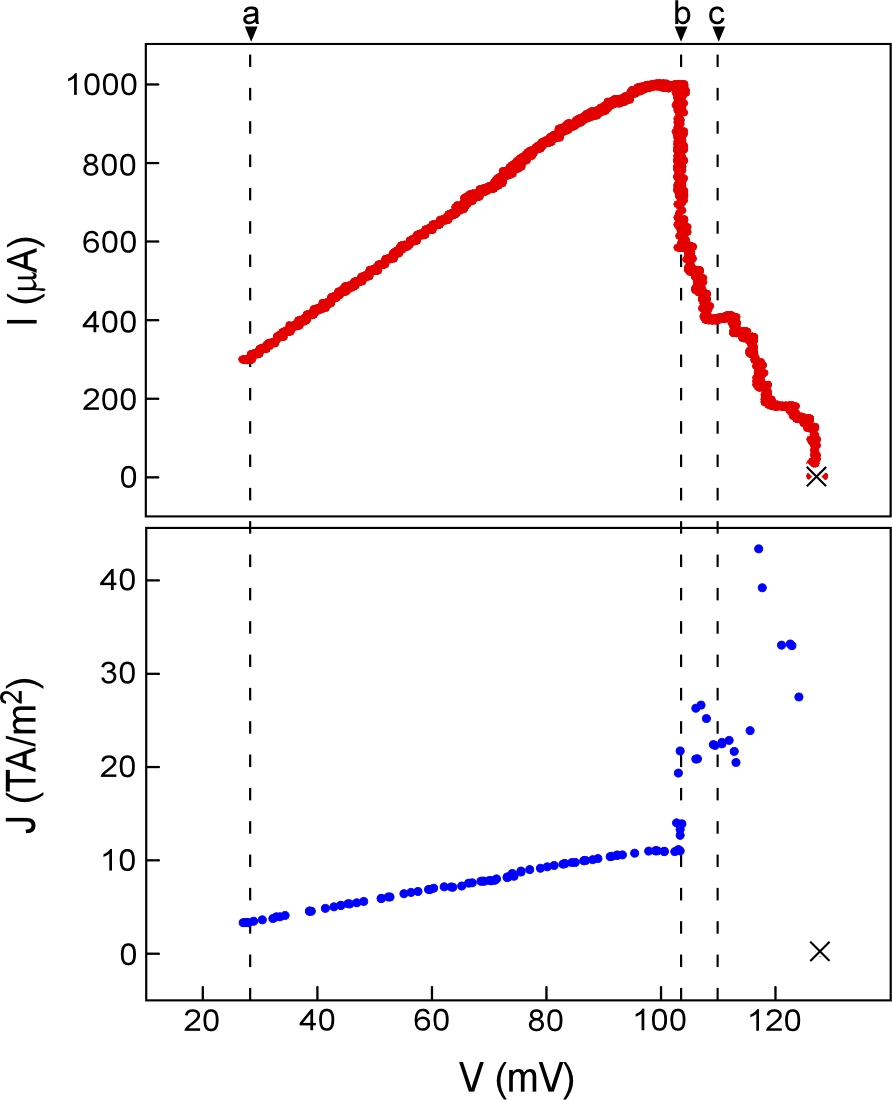


Figure S2


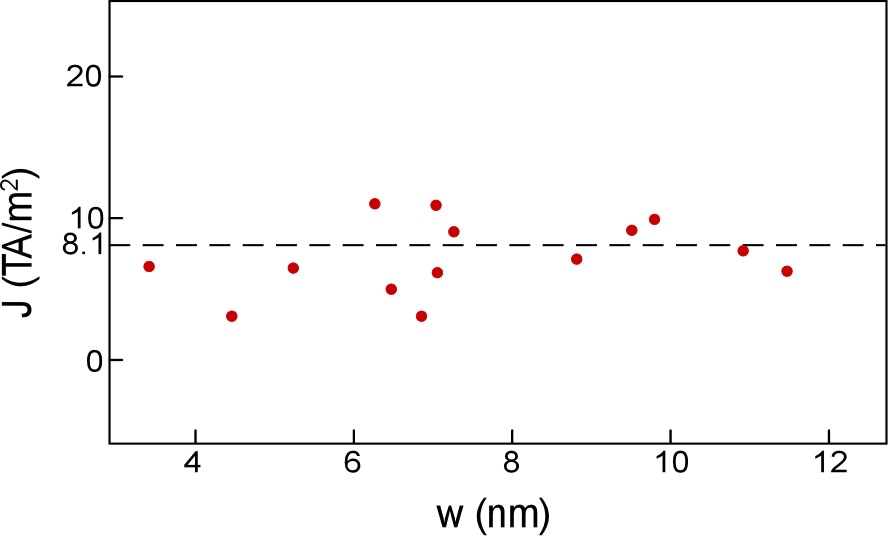


Figure S3


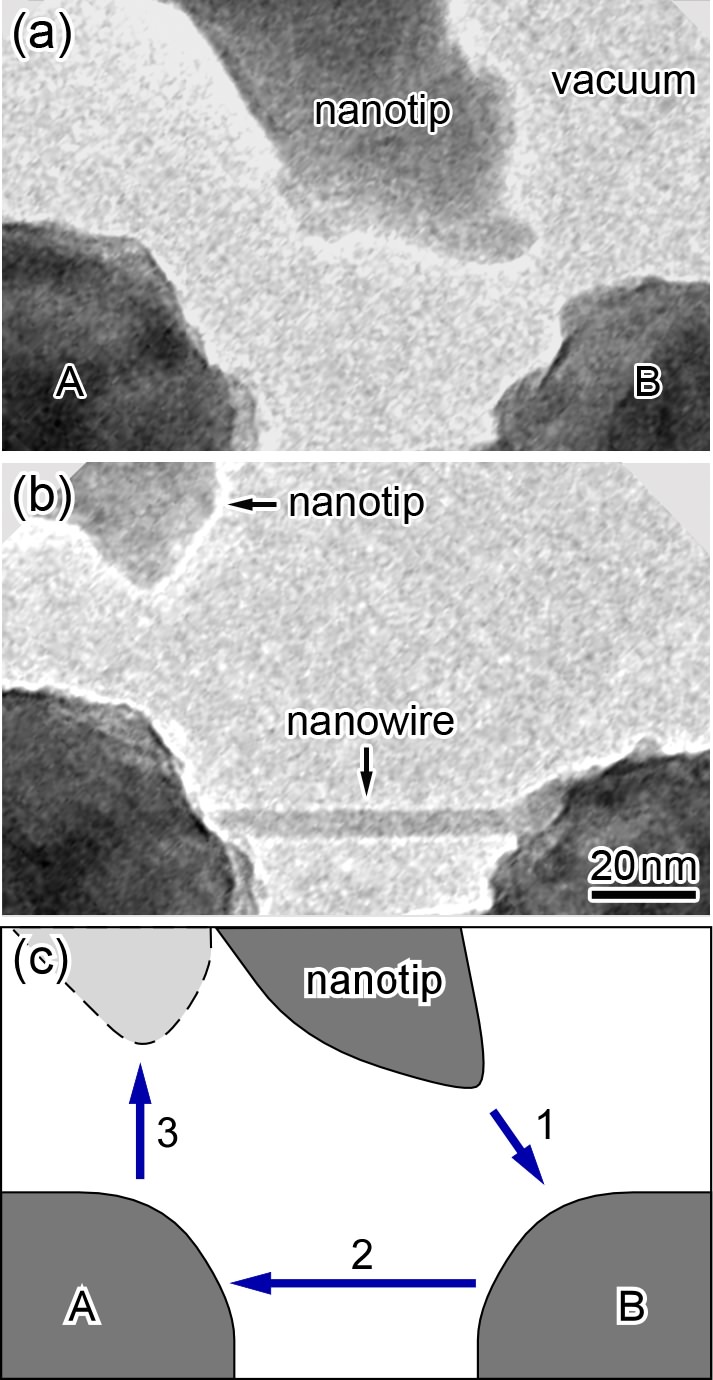


Figure S4
